# Insight into erythrocyte phospholipid molecular flux in healthy humans and in patients with acute respiratory distress syndrome

**DOI:** 10.1371/journal.pone.0221595

**Published:** 2019-08-27

**Authors:** Ahilanandan Dushianthan, Rebecca Cusack, Grielof Koster, Michael P. W. Grocott, Anthony D. Postle

**Affiliations:** 1 NIHR Southampton Biomedical Research Centre, University Hospital Southampton NHS Foundation Trust, Southampton, Hampshire, England, United Kingdom; 2 Integrative Physiology and Critical Illness Group, Clinical and Experimental Sciences, Sir Henry Wellcome Laboratories, Faculty of Medicine, University of Southampton, Southampton, Hampshire, England, United Kingdom; 3 Critical Care/ Anaesthesia and Perioperative Medicine Research Unit, University Hospital Southampton NHS Foundation Trust, Southampton, Hampshire, England, United Kingdom; Louisiana State University Health Sciences Center, UNITED STATES

## Abstract

Although the distribution of cellular membrane phospholipid composition is well characterised in human erythrocytes, in-vivo turnover and dynamic flux of phospholipids between plasma and erythrocytes in physiological and in particular during disease states are mostly unknown. Erythrocyte mass primarily consisted of lipids and phosphatidylcholine (PC) contributes to the significant proportion of phospholipid membrane composition. Esterified membrane PC can be utilised during pathological processes to generate pro and anti-inflammatory lipid mediators, which can contribute to the pathogenesis of acute respiratory distress syndrome (ARDS). In this study, utilising isotope labelling of choline and analytical methods with electrospray mass spectrometry (ESI-MS/MS), we characterised individual molecular composition and dynamic exchange of PC, sphingomyelins (SM) and lysophosphatidylcholines (LPC) between plasma and erythrocytes. In ARDS patients, there were significant alterations in PC molecular composition, coupled with a continuous loss of arachidonoyl-PC species over time. Infusion of *methyl-*D_9_-choline chloride resulted in enrichment of labelled choline into plasma PC and LPC via CDP-choline pathway with subsequent incorporation into erythrocyte PC. As expected, erythrocyte *methyl*-D_9_ PC enrichment is much slower than plasma. Patients had much faster and higher fractional enrichment of all PC and LPC molecules suggesting increased flux between plasma and erythrocytes. There was a particular pattern of incorporation, where the arachidonoyl-PC species achieved equilibrium with plasma rapidly and retained highest concentrations of enrichment compared to the other PC species. Increased enrichment of arachidonoyl-PC coupled with virtually no increase or depletion of its concentrations suggests the possibility of substrate donation for other cell types for the participation of eicosanoid biosynthesis during inflammatory conditions like ARDS. In summary, this study revealed an alerted pattern erythrocyte molecular phospholipid composition and flux in patients with acute respiratory distress syndrome and the pathological consequences of these changes needs further exploration.

## Introduction

Mammalian cell membranes consist of a mixture of glycerophospholipids and proteins. Phosphatidylcholines (PC) followed by phosphatidylethanolamines (PE) are the most abundant glycerophospholipids [1]. Relative molecular composition of membrane phospholipids can vary between cells and primarily depends on the specific cellular function. In addition to the structural role, membrane phospholipids are involved in cellular regulatory mechanisms [2]. Characteristics of membrane phospholipid composition and their dynamic turnover during various disease states define the cell’s ability to execute not only many immunological functions but also intracellular signal transduction, maintenance of cellular membrane integrity and hence cell survival [2, 3]. Except for erythrocytes, synthesis *de novo* via the CDP-choline pathway with sequential acyl-remodelling of fatty acids moieties by phospholipases and acyltransferases defines the final membrane PC composition in all mammalian cells [4].

Compared to other eukaryotic cells, erythrocytes are unique as they lack nuclei and mitochondria and are incapable of internal phospholipid biosynthesis. Despite their limited ability to synthesise lipids, more than 50% of erythrocyte membrane mass is made up by lipids and primarily consists of cholesterol, PC, PE, sphingomyelin (SM), phosphatidylserine (PS) and phosphatidylinositol (PI), neutral lipids and lysophosphatidylcholine (LPC) [5]. However, this distribution of lipids is not uniform throughout the membrane, and it appears that most of the PE and the PS are within the inner membrane and whereas the PC and SM are contained within outer leaflet [6]. Passive exchange between plasma and erythrocytes allows continuous renewal of erythrocyte membrane PC [7]. As a result, substrate availability from plasma may influence or alter the final membrane PC composition and could affect its ability to function and hence shorten its survival. Despite this complex molecular structure of the erythrocyte membrane, there is little exchange of other glycerophospholipids between plasma and erythrocytes [8].

Acute respiratory distress syndrome (ARDS) is a form of severe respiratory failure associated with systemic inflammation and multi-organ dysfunction [9]. The pathophysiology of ARDS is a complex process. There is overwhelming upregulated activation of systemic inflammatory response leading to alveolar epithelial and endothelial damage. Subsequently, the alveolar space is flooded by exudative oedema and plasma constituents [10]. Phospholipase-mediated breakdown of membrane phospholipids can generate pro-inflammatory mediators such as arachidonic acid metabolites. Elevated levels of these metabolites are identified in bronchoalveolar lavage fluid (BALF) and are at least partly implicated in the pathogenesis of ARDS [11]. The exact contribution of erythrocytes as donors of phospholipids to other inflammatory cells during the state of stress is not fully explored. Besides, the redistribution of phospholipids may have potential clinical consequences as the viability of the cell becomes compromised.

Moreover, inflammation is associated with anaemia and is often readily recognised in critical-illness and sepsis [12]. During inflammation, exposure to oxidative stress can lead to an influx of calcium ions and subsequent scrambling of the erythrocyte cell membrane [13]. The consequent externalisation of phosphatidylserine results in functional changes leading to microcirculatory alterations and subsequent removal of the cell by the reticuloendothelial system [14]. The erythrocyte membrane structure is dependent on the phospholipid composition, and any changes in the plasma composition may cause membrane asymmetry [15], which can induce morphological changes and ultimately lead to thrombogenic activation of erythrocytes [16]. Systemic inflammation and microcirculatory abnormalities are characteristic of ARDS and can be associated with an imbalance between pro- and anti-coagulant state [17]. Although lipid composition in erythrocytes is well documented, the dynamic phospholipid composition and flux during disease states such as ARDS have not been previously characterised.

Despite the existing wealth of information regarding erythrocyte static phospholipid compositions and flux kinetics of erythrocytes from in-vitro animal models, there are no human dynamic studies investigating parallel changes between erythrocytes and plasma *in-vivo*. Moreover, the variations in the exchange of individual phospholipid species between plasma and erythrocytes have not been previously investigated. Recently, *methyl-*D_9_ choline chloride has been used to investigate phospholipid flux *in-vivo* in the alveolar compartment to study surfactant biosynthesis in ARDS patients [18]. This method can also be used to investigate phospholipid flux in various cellular compartments [19]. However, this is the first study to investigate erythrocyte phospholipid turnover *in vivo* in a healthy human model and of a severe systemic global inflammatory condition. In this study, we investigated the static phospholipid composition of whole erythrocyte in healthy human volunteers and dynamic flux between plasma and erythrocytes. We then compared this with patients with ARDS over four days during their stay in our intensive care unit. We traced dynamic molecular enrichment patterns in plasma PC, lysoPC and SM using *methyl-*D_9_ choline chloride, a labelled precursor for PC synthesis via CDP-choline pathway and measured subsequent molecular PC arrangement in human erythrocytes. Electrospray ionisation mass spectrometry (ESI-MS/MS) enabled absolute molecular quantification of both plasma and erythrocyte phospholipid composition.

## Materials and methods

The study was approved by National Ethics Committee (10/WNo01/52) and Research and Development Department at University Hospital Southampton NHS Foundation Trust (CRI0244). Written consent was obtained from all participants. All patient and healthy volunteers were recruited between 2010 and 2014.

### Materials

*Methyl*-D_9_ choline chloride was from Cambridge Isotopes (CK Gases, Ibstock, UK); dimyristoylphosphatidylcholine (PC14:0_14:0), heptadecyllysophosphatidylcholine (LPC17:0) were from Avanti Polar Lipids (Alabaster, USA). Solvents (dichloromethane, methanol, butanol, NH_4_OH) of HPLC quality from Fisher Scientific, UK.

### Study population and *methyl*-D_9_-choline chloride infusion

Mechanically ventilated patients with ARDS (N = 8) were identified according to the American European Consensus Criteria (AECC) [20]. All of these patients had acute severe respiratory failure primarily related to intrinsic lung pathology such as pneumonia. All recruited patients were mechanically ventilated and enrolled within 72 hours of onset of ARDS. Patients had severe ARDS as measured by a mean PaO_2_/FiO_2_ ratio of 96 mmHg (range 63–138 mmHg) and a poor lung compliance of 30 cm of water. On average, patients were mechanically ventilated for 10 days with a total intensive care unit length of stay of 14 days.

We recruited healthy human volunteers (N = 5) without any prior medical conditions as controls. Once patients and controls were recruited, they were infused with 3.6 mg/kg of *methyl*-D_9_ choline chloride (Cambridge Isotopes) over a 3-hour period. Blood samples were collected in EDTA specimen bottles at baseline and 12, 24, 48, 72 and 96 in patients and 8, 24, 48, 72 and 96 hours in controls after *methyl*-D_9_ choline chloride infusion.

Healthy controls were not age-matched with patients (p = 0.001). There is no difference in total dose of *methyl-*D_9_ choline infused between patients and controls. The baseline characteristics and the doses of choline infused are presented in Table 1.

**Table 1:** Demographics of participants and the doses of *methyl*-D_9_ choline infused.

	Patients (N = 8)	Controls (N = 5)	p-value
Age [years in mean (range)]	63 (38–90)	25 (18–36)	0.001
Gender [male/female]	4/4	2/3	NA
Weight [kg]	78 (54–140)	78 (63–100)	0.98
Dose of *methyl*-D_9_ choline infused [mg]	280 (200–500)	270 (220–360)	0.83

### Lipid extraction

Erythrocytes were extracted from the packed cell pellet formed when whole blood was centrifuged at 400 x g at room temperature for 15 minutes. They were then washed with 0.9% saline (w/v) followed by repeated centrifugation twice prior to lipid extraction. Total lipid was extracted by modified Bligh and Dyer method [21]. Dimyristoyl PC (PC14:0_14:0) and LPC 17:0 were used as internal standards. The phospholipid fraction was extracted from aliquots of 100μl of erythrocyte packed cells or 100μl of plasma by dichloromethane: methanol: water (v/v 2:2:1). The recovered lipid rich lower phase was then died under nitrogen gas at 37°C and stored at -20°C for ESI-MS/MS analysis.

### Mass spectrometry and data extraction

Stored aliquots were dissolved in methanol: butanol: H_2_O: 25% NH_4_OH (6:2:1.6:0.4 v/v) and infused directly into a triple quadruple mass spectrometer with an electrospray interface (Waters, UK) at a rate of 8μl/min. The mass spectra were acquired in both positive (ES+) and negative (ES-) ionization modes with m/z range of 2–3000. Typical MS/MS conditions were produced with capillary 2.80 KV, cone -30 V, source temperature 150°C, collision energy of 30 eV and a scan time of 12 seconds for each ionization modes. Collision induced dissociation (CID) by tandem MS/MS (ESI-MS/MS) produced a diagnostic phosphocholine fragment ion (m/z 184), which enabled identification of PC, LPC, sphingomyelin by precursor scanning of m/z 184. *Methyl*-D_9_ PC, LPC and SM were identified by corresponding precursor scans of m/z193^+^. The data was extracted from MS spectra after smoothing, baseline subtraction and correction for isotopic reductions using Masslynx software (Version 4). This was subsequently exported into an excel spreadsheet and analyzed using an in-house Excel macro analyzer. Individual molecular species are denoted by the generic formula A:a_B:b, where A and B are numbers of acyl carbons, a and b are numbers of unsaturated double bonds, determined by fragmentation of representative samples in negative ionization, and the underscore indicating no assignment of *sn-1* or *sn*-2 position [22].

### Statistical analysis

The data is presented as mean ± standard error of mean (SEM). Single comparisons were analyzed by two-tailed student T-test and multiple comparisons by ANOVA of variance. A p value of <0.05 was assumed to be statistical significance. All statistical analyses were performed on GraphPad Prism (version 7).

## Results

### Erythrocytes maintain a distinct distribution of phospholipid composition compared to plasma

**Fig 1:**
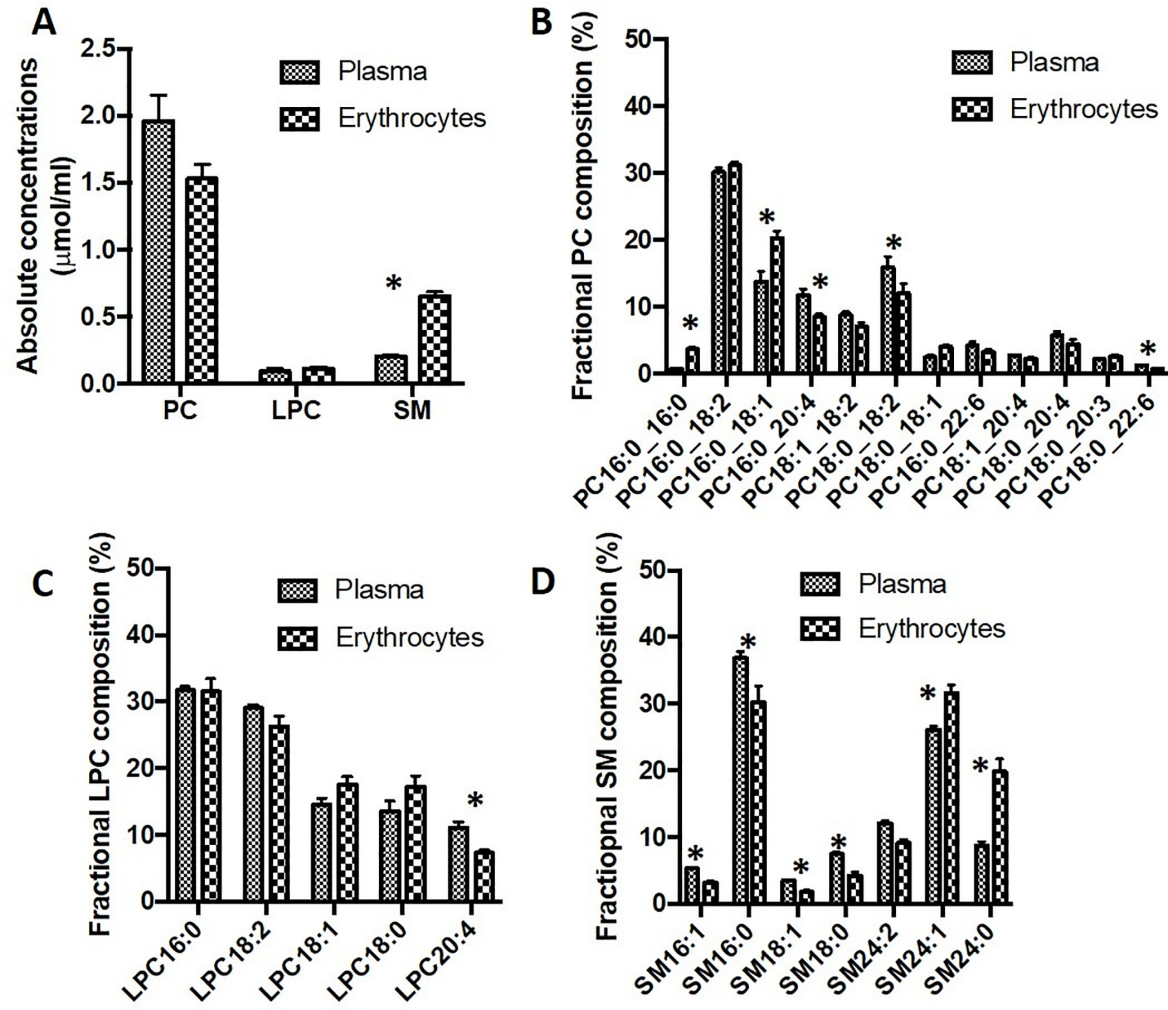
Choline-containing phospholipid composition in plasma and erythrocytes. A) Concentration of PC, LPC and SM in either plasma or packed erythrocyte cell pellet, expressed as μmol/ml. Comparison of molecular composition of PC (B), LPC (C) and SM (D) between plasma and erythrocytes, expressed as percentage of the total reported molecular species for that class. *P<0.05.

Applying specific precursor scans (P184^+^), ESI-MS/MS analysis demonstrated comparable concentrations of unlabelled PC and LPC in plasma and packed erythrocytes from healthy controls, but a significantly higher concentration of SM (Fig 1A). Erythrocytes maintained similar individual molecular species of PC, LPC and SM compared with plasma, but with significantly different distributions. Erythrocytes had fractionally more PC16:0_16:0 and PC16:0_18:1 compared to plasma and while polyunsaturated PC composition was generally lower (Fig 1B). The three main arachidonoyl PC species (PC16:0_20:4, PC18:1_20:4 and PC18:0_20:4), accounted for only 15% of erythrocyte PC compared to 21% in plasma. Similarly, erythrocyte LPC was also relatively depleted in the arachidonoyl species (LPC20:4) compared to plasma (Fig 1C). Sphingomyelin in plasma was dominated by palmitic (SM16:0, 37%), nervonic (SM24:1, 26%) and SM24:2 (12%) species, with higher fractional concentrations of lignoceric (SM24:0) and SM24:1 sphingomyelin (Fig 1D). These compositional differences indicate that, although choline-containing phospholipids exchange between erythrocytes and plasma, specific mechanisms must exist to regulate their final molecular specificities in erythrocytes.

Comparison between plasma and erythrocyte PC, LPC and SM compositions in ARDS patients indicates that previously reported alterations of plasma PC between ARDS patients and healthy volunteers are generally reflected in erythrocyte lipid compositions in the patient group (Table 2) [23]. ARDS patients exhibited significantly increased fractional compositions of monounsaturated (PC16:0_18:1) and disaturated (PC16:0/16:0) PC species in erythrocytes compared with healthy controls, with a similarly increased content of LPC18:1. These increases were at the expense of decreased contents of PC16:0_18:2, LPC18:0 and LPC18:2 and generally reflected relative plasma distributions between patients and controls. By comparison, there were no compositional differences between the major SM species between the two groups.

**Table 2:** Comparison of phospholipid composition between ARDS patients and controls in plasma and erythrocytes at enrolment.

Molecular species	Plasma			Erythrocytes		
Phosphatidylcholines	ARDS patients	Healthy humans	Ratio	ARDS patients	Healthy humans	Ratio
PC16:0_16:0	1.57 ± 0.28	0.79 ± 0.07	1.99	4.59 ± 0.24	3.70 ± 0.30	1.24
PC16:0_18:2	24.40 ± 1.12	30.16 ± 0.66	0.81	25.69 ± 1.19	31.17 ± 0.49	0.82
PC16:0_18:1	22.20 ± 1.13	13.81 ± 1.50	1.61	26.19 ± 1.40	20.24 ± 1.12	1.29
PC16:0_20:4	10.61 ± 1.15	11.78 ± 0.91	0.90	9.67 ± 1.12	8.47 ± 0.51	1.14
PC18:1_18:2	6.53 ± 0.45	8.81 ± 0.51	0.74	5.90 ± 0.34	7.07 ± 0.57	0.83
PC18:0_18:2	14.31 ± 0.80	15.87 ± 1.67	0.90	10.96 ± 0.36	12.00 ± 1.50	0.91
PC18:0_18:1	3.93 ± 0.47	2.47 ± 0.33	1.59	4.85 ± 0.32	4.01 ± 0.28	1.20
PC16:0_22:6	3.49 ± 0.55	4.28 ± 0.48	0.82	3.00 ± 0.48	3.25 ± 0.37	0.92
PC18:1_20:4	2.63 ± 0.20	2.76 ± 0.11	0.95	2.14 ± 0.19	2.21 ± 0.25	0.97
PC18:0_20:4	6.51 ± 0.50	5.79 ± 0.57	1.12	4.17 ± 0.40	4.48 ± 0.68	0.93
PC18:0_20:3	2.27 ± 0.47	2.20 ± 0.21	1.03	2.11 ± 0.19	2.65 ± 0.19	0.80
PC18:0_22:6	1.55 ± 0.24	1.27 ± 0.06	1.22	0.73 ± 0.11	0.75 ± 0.13	1.01
**Lysophosphatidylcholines**						
LPC16:0	28.43 ± 1.53	31.71 ± 0.57	0.90	34.20 ± 0.97	31.53 ± 1.93	1.08
LPC18:2	24.09 ± 1.17	29.06 ± 0.52	0.84	20.94 ± 1.15	26.31 ± 1.56	0.80
LPC18:1	21.49 ± 1.35	14.56 ± 0.92	1.48	25.15 ± 1.06	17.62 ± 1.10	1.43
LPC18:0	13.66 ± 1.11	13.60 ± 1.49	1.00	11.90 ± 0.63	17.28 ± 1.56	0.69
LPC20:4	12.33 ± 1.16	11.07 ± 0.94	1.11	7.81 ± 1.06	7.25 ± 0.46	1.08
**Sphingomyelin**						
SM16:1	5.68 ± 0.38	5.30 ± 0.14	1.07	4.37 ± 0.34	3.18 ± 0.23	1.37
SM16:0	41.28 ± 1.78	36.87 ± 1.00	1.12	34.91 ± 0.63	30.21 ± 2.48	1.15
SM18:1	3.29 ± 0.25	3.48 ± 0.05	0.95	2.20 ± 0.20	1.83 ± 0.23	1.20
SM18:0	7.94 ± 0.90	7.45 ± 0.33	1.07	4.99 ± 0.21	4.18 ± 0.54	1.19
SM24:2	11.34 ± 0.88	12.09 ± 0.36	0.94	7.72 ± 0.24	9.13 ± 0.55	0.85
SM24:1	25.08 ± 1.26	26.05 ± 0.57	0.96	29.49 ± 0.84	31.65 ± 1.18	0.93
SM24:0	5.38 ± 0.51	8.76 ± 0.57	0.61	16.32 ± 0.66	19.82 ± 1.95	0.82

Data presented as mean % composition (± standard error). The ratio is presented as patient’s composition (%) /control’s composition (%). Abbreviations: LPC, lysophosphatidylcholine; PC, phosphatidylcholine; SM, sphingomyelin.

**Fig 2:**
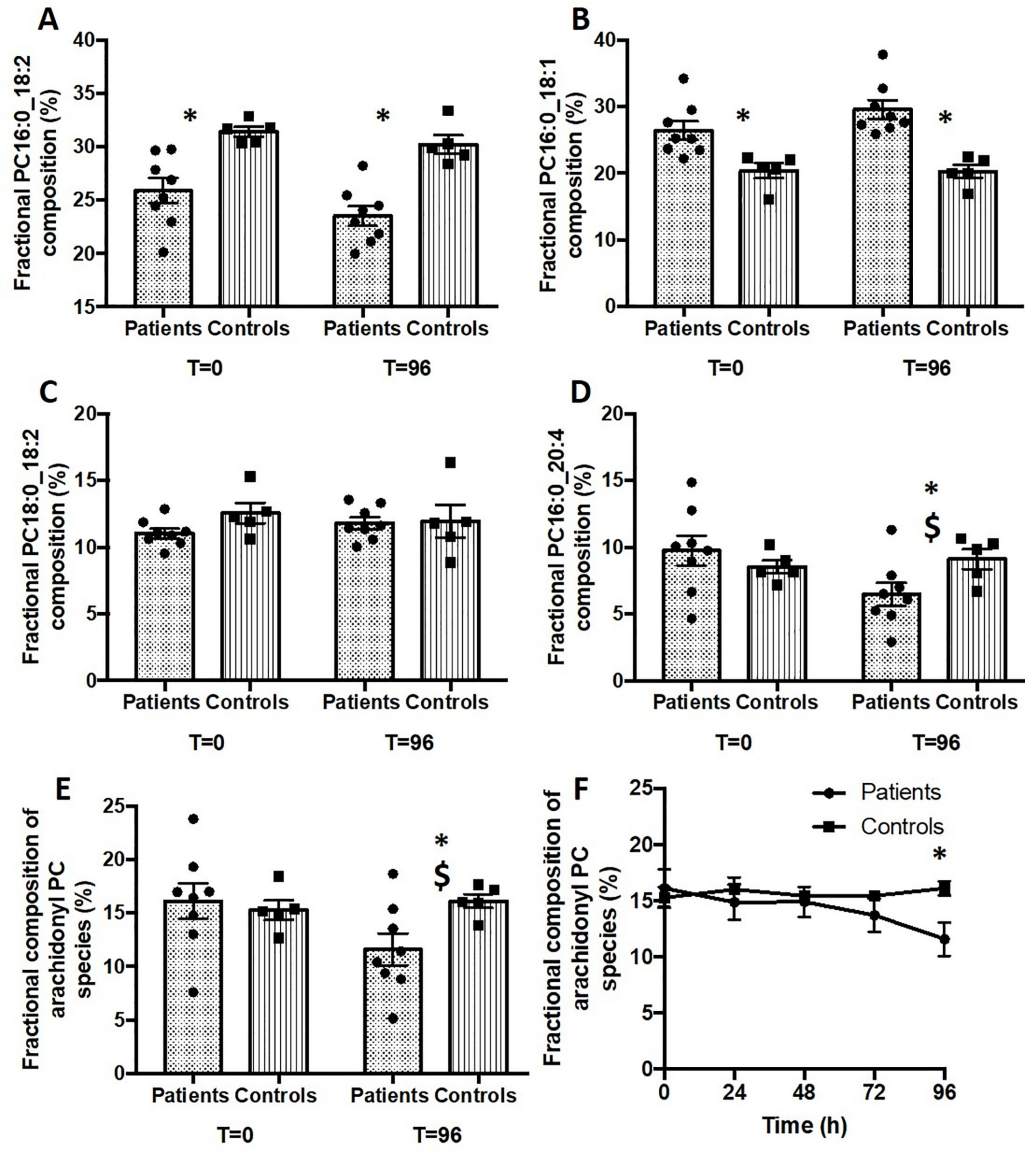
Compositional variation of major selected erythrocyte PC species between ARDS patients and healthy controls. **Results are shown as both grouped and individual data at baseline and 96 hours for A) PC16:0_18:2, B) PC16:0_18:1, C) 18:0_18:2 and D) 16:0_20:4 and E) total arachidonoyl-containing PC. Panel F details the overall gradual change with time for total arachidonoyl-PC.** *P<0.05 comparison between patients and controls. ^$^P<0.05 for comparison between 0 and 96 hours in ARDS patients.

Compositional differences of selected PC species in erythrocytes with time after recruitment are detailed in Fig 2. There were no changes to any PC species between T = 0 and T = 96 hours for the healthy control group, illustrated by these four species that combined contributed over 70% of total PC. While the fractional concentrations of PC 16:0_18:2 (Fig 2A), PC16:0_18:1 (Fig 2B) and PC18:0_18:2 (Fig 2C) did not change between T = 0 and T = 96 hours, the fractional concentration of erythrocyte PC16:0_20:4 in the ventilated ARDS patients was significantly lower at 96 h (6.5% total PC) compared with recruitment (9.8%) (Fig 2D).

These mean fractional concentrations mask considerable patient variability, demonstrated by the individual patient data in Fig 2. In contrast to the small variability for all species in the healthy control group, total arachidonoyl-PC exhibited a wide distribution at both time points (Fig 2E), with preservation of rank order such that by 96 hours arachidonoyl-PC species were lower for all patients. The variability of PC16:0_18:2 (Fig 2A) was intermediate between that of arachidonoyl-PC and the tighter distribution of PC16:0_18:1 (Fig 2B). The temporal trends illustrated in Fig 2D and 2E at 96 hours were reflected by a gradual decrease with time for total arachidonoyl-PC in ARDS patients (Fig 2F).

### The kinetics of phospholipid exchange between plasma and erythrocytes

**Fig 3:**
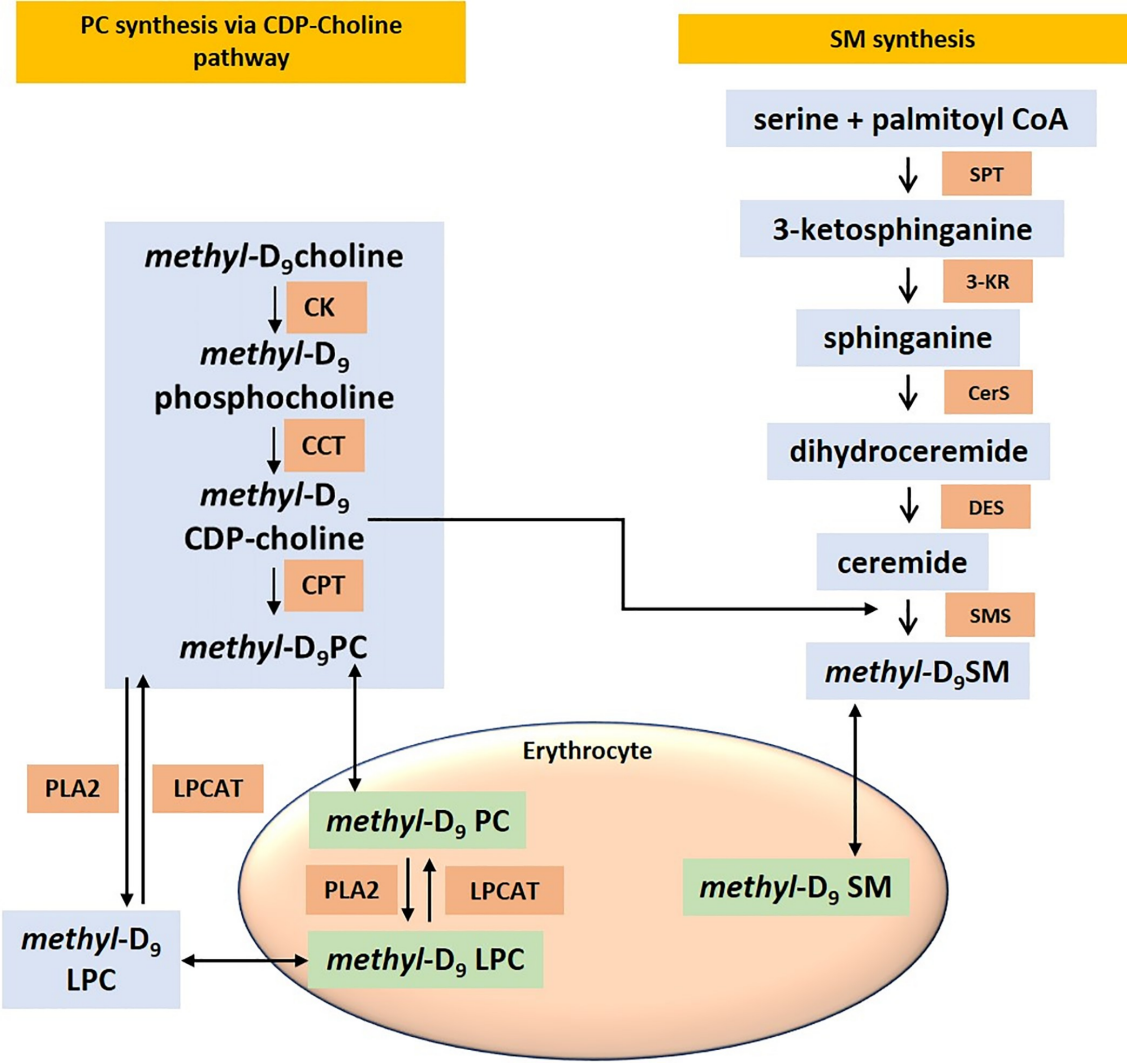
Schematic illustration of the incorporation of *methyl-*D_9_ choline in to the synthetic pathways of PC, LPC and sphingomyelin. Abbreviations: CK, choline kinase; CCT, CTP-phosphocholine cytidylyltransferase; CPT, choline phosphotransferase; PLA_2_, phospholipase A_2_; LPCAT, lysoPC acyltransferase; SPT, Serine palmitoyltransferase; 3-KR, 3- Ketosphinganine reductase; CerS, Ceramide synthase; DES, dihydroceramide desaturase; SMS, Sphingomyelin synthase.

Erythrocytes in theory should exchange all their phospholipids and fatty acids from plasma. Moreover, they have no enzymatic machinery to synthesize PC through either CDP-choline or PEMT pathways [24]. As a result, the final erythrocyte membrane composition is likely to depend on three possible mechanisms. Firstly, from the initial composition of PC synthesized by bone marrow derived reticulocytes. Secondly, from the exchange of phospholipids from plasma. Thirdly, acyl-remodeling of fatty acids and lipid exchange to and from plasma and other cell types (Fig 3). These mechanisms interact in relative proportions to maintain the flux and renewal of membrane PC in erythrocytes.

The phosphatidylcholines are synthesized in the liver by both CDP-choline and PEMT pathways and subsequently transported as lipoproteins in the plasma [25]. The incorporation of *methyl*-D_9_ choline into the labeled plasma fraction and later into the erythrocyte PC provides a novel mechanism to monitor PC exchange between these two highly phospholipid enriched compartments. We have previously demonstrated the specificity and kinetics of *methyl*-D_9_ choline into plasma PC in healthy controls and patients with ARDS [23]. The combination of these two parameters provides the mechanistic approach to quantify exchange of PC, LPC and SM between plasma and erythrocytes (Fig 3). The enrichment of *methyl*-D_9_ choline in phospholipid classes (PC, LPC, SM) was calculated from total *methyl*-D_9_ labelled species (P193) relative to the total unlabeled species (P184) from the following equation;

$$\%\;enrichment=\frac{P193}{\Sigma (P184+P193)}\times 100$$

**Fig 4:**
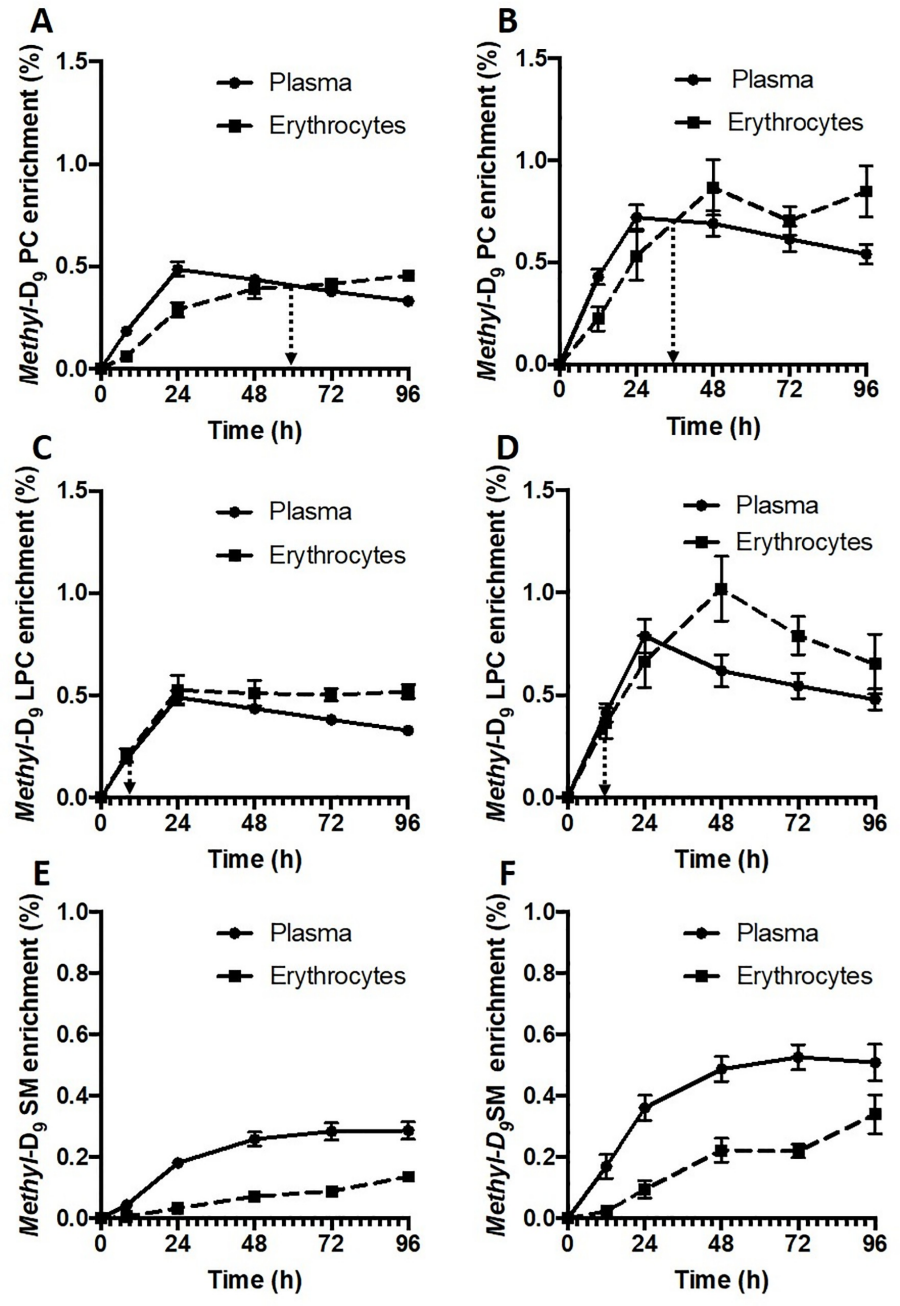
*Methyl-*D_9_ enrichment (%) of choline containing phospholipids in plasma and erythrocytes. A) Phosphatidylcholine in healthy controls, B) Phosphatidylcholine in ARDS patients, C) Lysophosphatidylcholine in healthy controls, D) Lysophosphatidylcholine in ARDS patients, E) Sphingomyelin in healthy controls and F) Sphingomyelin in ARDS patients. Dotted line represents the time when equilibrium achieved between plasma and erythrocytes.

There were remarkable differences in enrichment kinetics both between PC, LPC and SM classes and between patient and control groups (Fig 4). Erythrocyte *methyl*-D_9_ PC enrichment lagged behind that of plasma PC for healthy controls and achieved isotopic equilibrium at 60 hours (Fig 4A). In contrast, patient PC enrichment was consistently higher for both plasma and erythrocytes (Fig 4B) at all time points. In addition, the rate of exchange of labeled PC was much faster in the patient compared with the control group, with the time taken to reach isotopic equilibrium between plasma and erythrocyte PC decreasing from 60 to 36 hours (Fig 4A and 4B). As the exchange rate of unlabeled PC should be the same as that of the labelled PC, this equilibrium time represents the time when on average for all PC species, the plasma and erythrocyte pools reach equilibrium. The kinetics for exchange of labeled LPC was considerably more rapid than that of PC for both healthy controls (Fig 4C) and ARDS patients (Fig 4D), being at isotopic equilibrium by the earliest time points. Finally, not only was enrichment of the *methyl*-D_9_ label in SM lower than for either PC or LPC for both control (Fig 4E) and patient (Fig 4F) groups, but the rate of label exchange between plasma and erythrocytes was very slow and did not achieve isotopic equilibrium within the timescale of this study. These results indicate a selective pattern of phospholipid turnover in human erythrocytes during physiological state with variations in exchange rates in the order of LPC > PC > SM. These variations may be as a result of differences in physical properties, anatomical localization or due to the lack of flexibility in the exchange potential of that particular phospholipid in question. For example, the exchangeable proportion of total sphingomyelin pool in erythrocytes may be restricted to maintain cell membrane integrity. These results highlight the complexities of phospholipid exchange and renewal mechanisms in erythrocytes and suggest that they are highly metabolically active than just passive recipients of plasma phospholipids.

### The molecular specificity of phospholipid exchange between plasma and erythrocytes

Although there is existing literature in human and animal erythrocytes using *in-vitro* models of radioisotope ^14^C labelling of cholesterol and fatty acids or inorganic phosphorus ^32^P into phosphatic acid, this is the first study to investigate *in-vivo* exchange of individual molecular phospholipid species between plasma and erythrocytes in humans [7, 8, 24]. We quantified the *methyl*-D_9_ enrichment for all major PC, LPC and SM species accounting for >90% of their phospholipid group composition. There were considerable differences in rate of exchange of individual molecular phospholipid species between erythrocytes and plasma.

The kinetic exchange parameters for all reported lipid species are summarized for the control group in Table 3. The initial rate expressed as % of enrichment/ hour was consistently more rapid for all plasma PC species containing palmitate (16:0) than for the corresponding stearoyl (18:0)- containing species. For instance, initial rates of plasma *methyl*-D_9_ PC enrichment for PC16:0_18:1, PC16:0_18:2 and PC16:0_20:4 (respectively 0.030 ± 0.005, 0.022 ± 0.002 and 0.019 ± 0.001%/hour) were higher than those for PC18:0_18:1, PC18:0_18:2 and PC18:0_20:4 (respectively 0.019 ± 0.003, 0.015 ± 0.002, 0.012 ± 0.001%/hour). The maximum plasma enrichment was also highest for PC16:0_18:1 (0.74 ± 0.05%) which was followed by PC16:0_18:2 (0.55 ± 0.03%) and PC16:0_20:4 (0.49 ± 0.03%).

**Table 3:** Specificity of *methyl-*D_9_ PC enrichment (initial rate, maximum enrichment, equilibrium time between plasma and erythrocytes and time to maximum enrichment) for individual molecular PC, LPC and sphingomyelin species in healthy controls.

Individual Species	Initial rate (%/h)		Peak enrichment (%)			Isotopic equilibrium time (h)	Time to peak enrichment (h)	
	Plasma	Erythrocytes	Plasma	Erythrocytes	Ratio		Plasma	Erythrocytes
PC16:0_16:0	0.017 ± 0.009	0.003 ± 0.001	0.44 ± 0.05	0.30 ± 0.03	0.68	Not achieved	24 hours	96 hours
PC16:0_18:2	0.022 ± 0.002	0.010 ± 0.002	0.55 ± 0.03	0.56 ± 0.03	1.02	48 hours	24 hours	72 hours
PC16:0_18:1	0.030 ± 0.005	0.006 ± 0.001	0.74 ± 0.05	0.44 ± 0.04	0.59	84 hours	24 hours	96 hours
PC16:0_20:4	0.019 ± 0.001	0.016 ± 0.001	0.49 ± 0.03	0.73 ± 0.14	1.49	26 hours	24 hours	48 hours
PC18:1_18:2	0.019 ± 0.001	0.007 ± 0.002	0.45 ± 0.02	0.51 ± 0.03	1.13	61 hours	24 hours	96 hours
PC18:0_18:2	0.015 ± 0.002	0.006 ± 0.001	0.40 ± 0.02	0.32 ± 0.03	0.80	96 hours	48 hours	96 hours
PC18:0_18:1	0.019 ± 0.003	0.004 ± 0.001	0.45 ± 0.05	0.30 ± 0.05	0.67	Not achieved	24 hours	96 hours
PC16:0_22:6	0.017 ± 0.003	0.011 ± 0.002	0.43 ± 0.02	0.53 ± 0.04	1.23	24 hours	24 hours	48 hours
PC18:1_20:4	0.017 ± 0.003	0.010 ± 0.001	0.41 ± 0.02	0.51 ± 0.04	1.23	36 hours	48 hours	72 hours
PC18:0_20:4	0.012 ± 0.001	0.006 ± 0.001	0.36 ± 0.03	0.33 ± 0.03	0.91	8 hours	48 hours	96 hours
PC18:0_20:3	0.010 ± 0.001	0.003 ± 0.001	0.36 ± 0.01	0.21 ± 0.03	0.58	Not achieved	72 hours	96 hours
PC18:0_22:6	0.008 ± 0.001	0.004 ± 0.001	0.21 ± 0.02	0.22 ± 0.04	1.05	80 hours	48 hours	96 hours
LPC16:0	0.006 ± 0.0002	0.028 ± 0.001	0.57 ± 0.04	0.67 ± 0.06	1.17	< 8 hours	24 hours	24 hours
LPC18:2	0.008 ± 0.0005	0.014 ± 0.003	0.44 ± 0.04	0.55 ± 0.06	1.25	36 hours	24 hours	96 hours
LPC18:1	0.008 ± 0.0004	0.019 ± 0.001	0.65 ± 0.05	0.54 ± 0.07	0.83	40 hours	48 hours	48 hours
LPC18:0	0.015 ± 0.0010	0.019 ± 0.004	0.39 ± 0.03	0.59 ± 0.04	1.51	< 8 hours	72 hours	72 hours
LPC20:4	0.009 ± 0.0005	0.029 ± 0.002	0.39 ± 0.03	0.72 ± 0.26	1.85	< 8 hours	48 hours	24 hours
SM16:1	0.005 ± 0.001	0.005 ± 0.0007	0.33 ± 0.03	0.45 ± 0.05	1.36	36 hours	72 hours	72 hours
SM16:0	0.005 ± 0.001	0.003 ± 0.0001	0.26 ± 0.01	0.29 ± 0.03	1.11	88 hours	96 hours	96 hours
SM18:1	0.006 ± 0.002	0.003 ± 0.0006	0.29 ± 0.01	0.30 ± 0.02	1.03	45 hours	96 hours	96 hours
SM18:0	0.006 ± 0.001	0.0013 ± 0.0002	0.29 ± 0.03	0.11 ± 0.01	0.38	Not achieved	48 hours	96 hours
SM24:2	0.006 ± 0.001	0.0005 ± 0.0001	0.35 ± 0.02	0.08 ± 0.02	0.23	Not achieved	96 hours	96 hours
SM24:1	0.005 ± 0.001	0.0005 ± 0.0001	0.38 ± 0.01	0.06 ± 0.01	0.16	Not achieved	96 hours	96 hours
SM24:0	0.005 ± 0.001	0.0004 ± 0.0001	0.28 ± 0.04	0.05 ± 0.01	0.18	Not achieved	96 hours	96 hours

Data presented as mean ± standard error.

**Fig 5:**
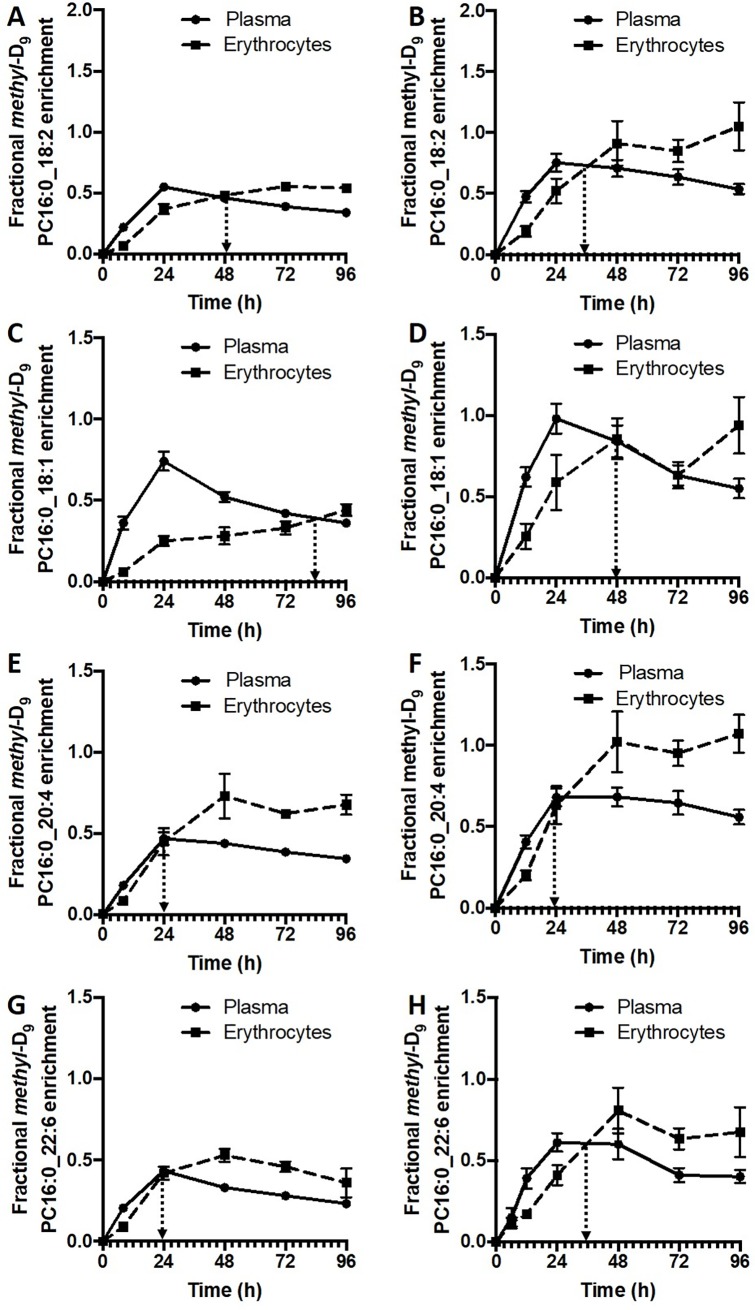
Molecular specificity of *methyl-*D_9_ PC enrichment (%) between plasma PC and erythrocytes. Comparison between heathy controls {PC16:0_18:2 (A), PC16:0_18:1 (C), PC16:0_20:4 (E) and PC16:0_22:6 (G)} and ARDS patients {PC16:0_18:2 (B), PC16:0_18:1 (D), PC16:0_20:4 (F) and PC16:0_22:6 (H)}. Dotted line represents the time when equilibrium achieved between plasma and erythrocytes.

In contrast, erythrocytes from control subjects demonstrated a selective *methyl*-D_9_ PC enrichment pattern that was very different from that of plasma. For instance, the initial rate of incorporation for PC16:0_20:4 into erythrocytes (0.016 ± 0.001%/hour) was more rapid than that for PC16:0_18:1 and PC16:0_18:2 (respectively 0.006 ± 0.001 and 0.010 ± 0.002%/hour), despite the reverse order of their enrichment rates in plasma. The maximal erythrocyte *methyl-*D_9_ enrichment was much lower for PC16:0_18:1, accounting for only 60% of plasma maximum enrichment. In contrast, maximal erythrocyte enrichments of PC16:0_20:4 and PC16:0_22:6 were 150% and 120% of their plasma counterparts respectively. However, maximal enrichment was similar for PC16:0_18:2 for both plasma and erythrocytes. Moreover, the erythrocyte *methyl*-D_9_ enrichment of individual PC species equilibrated with plasma at different time points (Table 3 and Fig 5). For three PC molecules (PC16:0_16:0, PC18:0_18:1 and PC18:0_20:3) erythrocyte *methyl*-D_9_ enrichment did not achieve equilibrium with plasma within the duration of this study period. Faster equilibrium times were noted for PC species containing polyunsaturated fatty acids, in the order of PC18:0_20:4 (8 hours), PC16:0_22:6 (24 hours), PC16:0_20:4 (26 hours) and PC18:1_20:4 (36 hours). This suggests that there is a selective uptake and turnover of polyunsaturated fatty acid PC molecules in erythrocytes (Table 3).

The overall and individual erythrocyte flux of LPC was much faster than that of plasma. The molecular *methyl-*D_9_ LPC enrichment was variable between plasma and erythrocytes for individual species, where LPC20:4 followed by LPC16:0 and LPC18:0 showed much faster enrichment rate and quicker equilibrium (< 8 hours) with plasma. For all LPC species, the erythrocyte *methyl-*D_9_ enrichment achieved equilibrium with plasma much earlier than that of PC species. This finding is suggestive of rapid exchange of LPC in erythrocytes, particularly of arachidonoyl-LPC (LPC20:4). Erythrocyte sphingomyelin molecular kinetics also demonstrated significant variability between molecular species. Although much slower than PC and LPC, SM16:1, SM16:0 and SM18:1 enrichment eventually achieved similar maximal enrichments compared with plasma. However, *methyl-*D_9_ choline enrichments into very long chain fatty acid sphingomyelins (SM 24:2, SM 24:1 and SM 24:0) were much lower and did not achieve equilibrium with plasma during the study duration (Table 3).

**Fig 6:**
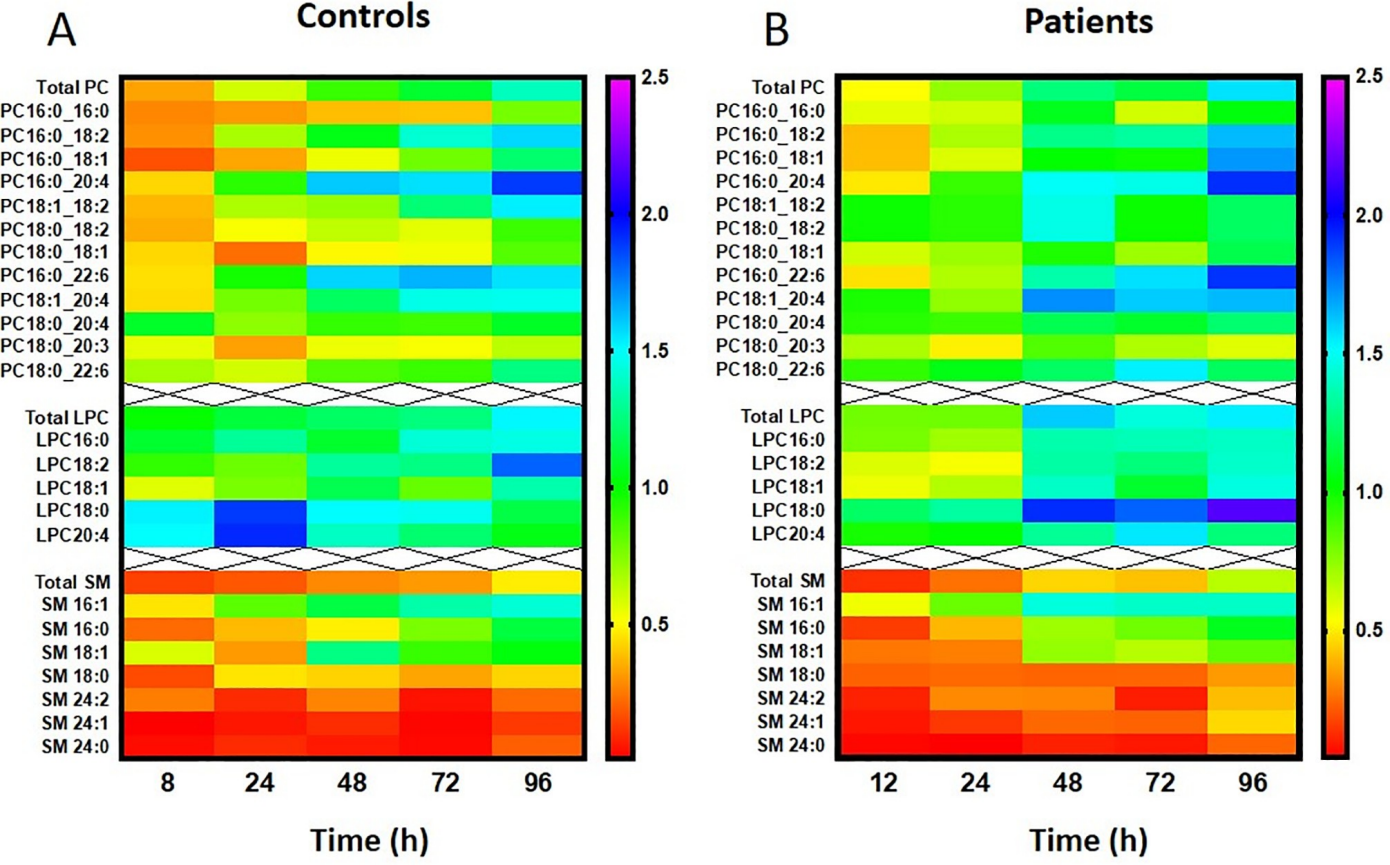
Molecular specificity of erythrocyte *methyl-*D_9_ PC, LPC and sphingomyelin enrichment over time between healthy controls and ARDS patients. Each color bar represents the percentage of fractional *methyl-*D_9_ enrichment (%) of the total phospholipid composition.

The kinetics of PC exchange between plasma and erythrocytes was considerably more rapid in ARDS patients compared to the control group. Fig 5 details comparative enrichment profiles of *methyl*-D_9_ PC of four major PC species for both patient and control groups. This effect was greatest for PC16:0_18:2 (Fig 5A and 5B) and PC16:0_18:1 (Fig 5C and 5D), with equilibrium times decreasing from 48 to 30 hours and from 84 to 48 hours respectively for ARDS patients compared with control subjects. By contrast, the rapid equilibration of polyunsaturated-containing between plasma and erythrocytes in the control group was not decreased in ARDS patients (Fig 5E, 5F, 5G and 5H). An overall comparison of label enrichments of all reported PC, LPC and SM species (Fig 6) demonstrates that, while enrichments of the majority of PC species were enhanced in patients compared with controls, this effect was less apparent for LPC species and was absent from SM species, for which the enrichments remained low for both groups.

## Discussion

Eukaryotic cellular lipid distribution consists of several hundreds of different types and varying combinations of glycerophospholipid species. This diversity of phospholipid composition depends on the cellular type and its function. Despite this diversity, there is a relative consistency in cellular phospholipid composition, vitally important to maintain not only the membrane integrity, but also to preserve specific cellular functions. In general, cellular phospholipids are distributed asymmetrically within membranes and continuously recycled and remodeled by the processes of biosynthesis, exchange and deacylation/reacylation mechanisms [26]. Compared to other mammalian cells, erythrocytes have limited capacity to synthesize phospholipids *de-novo* and as a result, they greatly depend on the availability of plasma lipids to maintain membrane integrity and function [27]. Alterations in erythrocyte phospholipid composition and turnover has been well documented in many diseases including sepsis [28], diabetes mellitus [29] and neuropsychiatric disorders [30] and are thought to be detrimental to the cell survival. Despite the extensive literature on erythrocyte phospholipid metabolism, human *in-vivo* mechanistic studies investigating dynamic phospholipid flux and exchange between erythrocytes and plasma are lacking, particularly in pathological conditions. Using non-radioactive isotope labelling of choline, which is a precursor for PC biosynthesis, in combination with ESI-MS/MS analytical methods, this is the first study to evaluate molecular specificity of erythrocyte phospholipid exchange in healthy humans and patients with refractory hypoxic respiratory failure.

The configuration of phospholipid subclasses revealed asymmetrical distribution of molecular species. ARDS patients demonstrated significant changes in their molecular arrangement predominantly in PC molecular composition. We observed a reduced proportion of linoleoyl and increased levels oleoyl composition in ARDS patients with no significant differences in polyunsaturated species at enrolment. The clinical implications of this linoleoyl deficit is not clear but may have been as a result of nutritional deficiencies. As erythrocytes have a long life span up to 120 days, their membrane composition has been evaluated as a surrogate for nutritional assessment, particularly reflecting the dietary intake of fatty acids. We did not control for any dietary intake as these patients were critically ill and supported by artificial enteral nutrition. However, we enrolled all patients within 72 hours of onset of ARDS and the mean duration between hospital admission and *methyl*-D_9_ choline chloride infusion was 1.9 days (range 0–4). For this reason, it is unlikely that the compositional changes noted on day 0 are related to intensive care nutritional interventions. However, it may reflect a chronic nutritional deficiency of ARDS patients prior to the hospital admission.

Although the erythrocyte PC composition was similar to that of plasma, erythrocytes had greater fractional compositions of PC16:0_16:0 and PC16:0_18:1 with lesser amounts of PC16:0_20:4 and PC18:0_18:2. This temporal relationship between erythrocytes and plasma was relatively maintained also by patients, despite the significant increase in PC16:0_18:1 from both tissues. The reason for this specificity is uncertain but is probably a reflection of surrounding plasma composition and exchange rates of this molecular PC between plasma and erythrocytes.

To understand the dynamic flux of molecular PCs further, we infused deuterium labelled choline chloride (*methyl*-D_9_ choline chloride) over a 3-hour period in both groups. This is used to synthesize PC through the CDP-choline pathway by plasma membrane and subsequently incorporated by bidirectional exchange into erythrocytes. Compared with healthy volunteers, ARDS patients had increased *methyl*-D_9_ PC enrichment in both plasma and erythrocyte fractions. There was a 60% increase in total plasma enrichment in patients, which was maintained throughout the study period. Erythrocyte enrichment was slower than that of plasma for both groups and in controls, the equilibrium between plasma and erythrocyte being achieved around 60 hours after the *methyl*-D_9_ choline chloride infusion. After this time point, as there was a steady decline in plasma coupled with a continued increase in erythrocyte *methyl-*D_9_ PC enrichment. This slower turnover of erythrocytes phospholipids has been demonstrated before in animal experiments [7]. The *methyl-*D_9_ PC enrichment between plasma and erythrocytes achieved its equilibrium at much earlier time point (36 hours) in patients, suggesting increased flux of exchange of phosphatidylcholines between plasma and erythrocytes. The underlying reason for this enhanced exchange in ARDS patients may be due to an adaptation to preserve membrane integrity in response to the severe inflammation characteristic of these patients.

The molecular specificity of fractional enrichment showed significant variation between individual PC species. For instance, the PC species with the arachidonoyl moiety (PC16:0_20:4, PC18:0_20:4 and PC18:0_20:4) equilibrated much faster with plasma than other PC molecules. By contrast, PC16:0_18:1 *methyl-*D_9_ enrichment in controls was very slow compared with plasma and achieved its equilibrium at 84 hours after *methyl*-D_9_ choline infusion. However, patient’s erythrocytes showed much faster equilibrium time points with plasma for all PC molecular species. This suggests that in physiological states, there is a specific molecular pattern of incorporation into erythrocyte membrane, which can be alerted and prioritised selectively in pathological conditions depending on cellular demand.

Specific to ARDS patients, there was a continued loss of arachidonoyl-PC species, which became more significant at day 4. Despite the noticeable increase in turnover of the arachidonoyl PC moiety in patients, the fractional composition of newly labelled arachidonoyl PC remained lower than that of controls with continued depletion of measured endogenous unlabelled fraction over time. This highlights a selective deficiency in the maintenance of concentrations of arachidonoyl-PC moiety in erythrocytes. It also suggests that the erythrocytes are involved in active participation of phospholipid metabolism and it is possible that they may act as a reservoir for PC supply and play a crucial role in the substrate provision for other cell types.

The LPC composition and fractional *methyl*-D_9_ enrichment also reflected that of the PC fraction, where there was a significant increase in LPC18:1 with lower concentrations of LPC18:2 in patients. Turnover of LPC is much faster than the PC fraction in both groups. However, patients had doubled the amount of *methyl*-D_9_ LPC enrichment in erythrocytes at peak enrichment, followed by a rapid decline. This suggests not only there is increased incorporation of LPC but also there is increased usage. This distinctive rapid erythrocyte *methyl*-D_9_ LPC enrichment suggests that LPC in erythrocytes must be derived from two separate mechanisms. It is possible, that the labelled LPC in erythrocytes are directly exchanged from the plasma as well as from hydrolysis of labelled erythrocyte PC. Furthermore, the similarities in the enrichment pattern between plasma PC and LPC indicates that plasma LPC is generated primarily in parallel with PC from the liver than as a result of acyl-remodeling mechanisms. As far as we know, this is the first study to evaluate *in-vivo* dynamic exchange of LPC from human erythrocytes in both physiology and disease states.

## Conclusions

We have demonstrated the alterations in erythrocyte composition between ARDS and healthy controls. Despite the similarities between plasma and erythrocyte, there were striking differences in PC composition with loss of arachidonic acid-based PC species. This was coupled with increased turnover of both PC and LPC labelled fraction. Despite this increase in turnover, it was not sufficient enough to maintain concentrations of selective PC molecules such as PC16:0_18:2 and PC16:0_20:4. This highlights that there is increased usage and the dynamic nature of phospholipid flux in ARDS patients. This is the first study to comprehensively assess lipid profiling and flux of choline containing phospholipids between plasma and erythrocytes in humans in physiological and disease states. Further studies are needed to evaluate the impact on clinical consequences with subsequent correlations with downstream arachidonic acid metabolites and erythrocyte life span.

## Supporting information

S1 Data (XLSX)

## Abbreviations

AECC: American European Consensus Criteria
ARDS: acute respiratory distress syndrome
BALF: bronchoalveolar lavage fluid
CDP: cytidine diphosphate
EDTA: ethylenediaminetetraacetic acid
ESI-MS/MS: electrospray ionisation mass spectrometry
ICU: intensive care unit
LPC: lysophosphatidylcholine
MD: mean difference
PC: phosphatidylcholine
PE: phosphatidylethanolamine
PG: phosphatidylglycerol
PI: phosphatidylinositol
PS: phosphatidylserine
SEM: standard error of mean
SM: sphingomyelin
